# Modulation of metabolic pathways and EndMT inhibition by a traditional Chinese herbal formula in the treatment of high-risk infections

**DOI:** 10.3389/fcimb.2024.1497965

**Published:** 2024-11-15

**Authors:** Hailing You, Kailai Bu, Liping Chen, He Sun, WangAo Suyang, Gaofei Feng, Yuan Chen

**Affiliations:** ^1^ Department of Oncology, Shenzhen Hospital of Beijing University of Chinese Medicine (Longgang), Shenzhen, China; ^2^ Guangzhou University of Chinese Medicine, Department of Dermatology, Guangdong Provincial Hospital of Chinese Medicine, Guangzhou, China; ^3^ Department of Obstetrics and Gynecology, Shenzhen Hospital of Beijing University of Chinese Medicine (Longgang), Shenzhen, China; ^4^ Department of Preparation, Shenzhen Hospital of Beijing University of Chinese Medicine (Longgang), Shenzhen, China; ^5^ Shenzhen SMQ Group Medical Laboratory, Shenzhen, China; ^6^ Department of Physical Examination, Shenzhen Hospital of Beijing University of Chinese Medicine (Longgang), Shenzhen, China

**Keywords:** combination of Chinese herb, HPV infection, metabolomics, endothelialto-mesenchymal transition (EndMT), traditional Chinese medicine

## Abstract

**Background:**

Herbal products have long been utilized as remedies for various disease conditions, including infections. This study investigates the therapeutic mechanism of a traditional Chinese herbal combination in treating high-risk HPV infections.

**Methods:**

The herbal formula was prepared using common herbs: dry Millettia speciosa, Guanzhong (a spermatophyte), Sarsaparilla, White Fruit, and Cockscomb Flower. Eight female patients diagnosed with high-risk HPV were enrolled from January to September 2023 at Shenzhen Hospital of Beijing University of Traditional Chinese Medicine. Cervicovaginal secretions were collected before and after treatment with the herbal remedy and analyzed using non-targeted metabolomics techniques. *In vitro* studies were conducted using HeLa cells to determine the optimal effective concentration of the remedy, assessed via the CCK8 method. The proliferation and migration of HeLa cells were evaluated using Transwell assays. Quantitative PCR was employed to measure mRNA levels of endothelial-to-mesenchymal transition (EndMT) markers, including VE-Cadherin, eNOS, α-SMA, and Snail.

**Results:**

*In vivo*, significant alterations in cervicovaginal secretion metabolites post-treatment were observed through PCA, OPLS-DA, and volcano plot analyses. KEGG enrichment analysis highlighted crucial signaling pathways such as arginine and proline metabolism, purine metabolism, glycerophospholipid metabolism, and phenylalanine metabolism, indicating the herbal combination’s systemic effects on patients. *In vitro* experiments demonstrated a dose-dependent reduction in HeLa cell proliferation and migration, confirmed by scratch and Transwell assays. Additionally, qPCR analysis revealed down-regulation of α-SMA and Snail, and up-regulation of VE-Cadherin and eNOS, suggesting inhibition of EndMT in HeLa cells.

**Conclusion:**

The traditional Chinese herbal combination modulates key metabolic pathways *in vivo* and inhibits EndMT *in vitro*, while reducing HeLa cell proliferation and migration. These findings highlight its potential as a therapeutic approach for managing HPV infections, bridging traditional practices with scientific research.

## Introduction

Human papillomavirus (HPV), a double-stranded cyclic DNA virus, has been strongly associated with the development of cervical cancer, particularly through persistent high-risk infections, with an HPV-positive rate exceeding 80% in cervical cancer patients (Barra et al., 2019; Guo et al., 2022). HPV types 16 and 18 are among the most prevalent high-risk strains globally, responsible for approximately 70% of cervical cancer cases worldwide (Ahmed et al., 2017). Annually, over 100,000 new cases of cervical cancer are reported in China alone, underscoring the significant role of high-risk HPV infection as a contributing factor. Addressing HPV infection is therefore crucial for the prevention and treatment of cervical cancer, necessitating intensified research into effective anti-HPV drugs to facilitate early intervention and slow disease progression. Traditional Chinese Medicine (TCM), renowned for its long history of treating gynecological conditions, has shown promise in improving outcomes in HPV-positive patients. For instance, clinical trials have demonstrated that certain TCM formulations not only alleviate symptoms but also promote the clearance of HPV infection and enhance immune responses. Notably, high-risk HPV strains like 16 and 18 have been a particular focus of these studies due to their aggressive nature and association with cancer progression.Studies on treatments like Fufang Yunzhi Ganoderma Lucidum (FYG) capsules, another herbal formula, have reported increased HPV clearance rates and improvement in cervicovaginal secretion quality (Gençtürk et al., 2024). Moreover, TCM-based therapies, including Kangfuxin solution, have been shown to accelerate HPV conversion and reduce recurrence rates in patients with cervical intraepithelial neoplasia. DaiNing Decoction is a herbal formula comprising Niu Da Li, Guan Zhong, Sarsaparilla, Bai Guo, and Cocklebur, known for its therapeutic effects in clearing heat, eliminating toxins, dispersing blood stasis, tonifying deficiencies, inducing dampness, and stopping bleeding. Banding Tang, a formula developed by the Shenzhen Hospital of Beijing University of Chinese Medicine based on clinical experience, has been clinically proven to effectively improve cervicovaginal secretions and promote HPV conversion in cervical cancer patients. However, the underlying mechanism of its action remains unclear. Metabolomics, which encompasses the assembly of biochemical reactions in organisms and serves as the material basis for sustaining life, is a valuable tool for studying various life processes. It enables tracking of disease progression, formulation of rational management plans, identification of drug targets for new drug development, and large-scale screening of new biomarkers for early prediction, diagnosis, and classification of diseases (Walboomers et al., 1999; Akbari et al., 2023). Consequently, metabolomics is an essential component of precision medicine, offering important technological means for research. Metabolomics can be divided into non-targeted and targeted metabolomics based on specific research objectives (Koal and Deigner, 2010; Hocher and Adamski, 2017). Non-targeted metabolomics involves the use of ultra-high-performance liquid chromatography-tandem high-resolution mass spectrometry (UPLC-MS/MS) to detect dynamic changes in all small molecule metabolites before and after stimulation or perturbation in cells, tissues, organs, or organisms, without any bias. This approach allows extensive scanning of metabolites and screening of differential metabolites through bio confidence analysis, followed by metabolite identification and pathway analysis to elucidate the underlying physiological mechanisms of the observed changes (Tokarz et al., 2017). By applying non-targeted metabolomics, we aim to explore the complex molecular interactions and pathways influenced by DaiNing Decoction, particularly in the context of high-risk HPV infection and associated cellular changes. To provide insights into the intrinsic mechanism of DaiNing Decoction, this study aims to investigate the mechanism of action of Banding Tang in treating HPV infection using non-targeted metabolomics technology based on liquid chromatography-mass spectrometry (LC-MS).

## Methods

The present study received ethical approval from the Institutional Review Board (IRB) of Shenzhen Hospital, Beijing University of Traditional Chinese Medicine. All procedures and protocols were conducted in accordance with the principles outlined in the Declaration of Helsinki and other relevant ethical guidelines. Informed consent was obtained from all participants before their inclusion in the study. Confidentiality of participant information was strictly maintained throughout the study duration.

### Participant selection and screening

Female patients diagnosed with high-risk HPV persistent infection at Shenzhen Hospital of Beijing University of Traditional Chinese Medicine between January 2023 and September 2023 were included. Diagnosis of HPV infection followed the 2022 edition of the Cervical Cancer Diagnosis and Treatment Guidelines, combining ThinPrep Cytology Test (TCT), HPV testing, colposcopy, pathology, imaging, and tumour markers exams. Inclusion criteria comprised pathologically or cytologically confirmed HPV infection in women aged 18 to 60 years, with full informed consent. Exclusion criteria included concurrent bacterial vaginitis, mycoplasma and chlamydia infections, mycobacterial infectious vaginitis, and trichomonas vaginitis. Additionally, patients experiencing serious adverse events, complications, or physiological changes during the trial, self-withdrawal, loss of visit, incomplete information, or medication use outside the prescribed range were excluded. Pre-treatment, cervical and vaginal secretions were collected for metabolomics, vaginal flora, and HPV testing. Post-treatment, the same samples were collected for comparison.

### Preparation of DaiNing (Harb Combination) Decoction

DaiNing Decoction consisted of 6kg of *Millettia speciosa* commonly known as Niu Dali, 2.7kg of one spermatophyte namely Guanzhong, 9kg of *Sarsaparilla*, 1.5kg of *White Fruit*, and 4.5kg of *Cockscomb* Flower. The ingredients were mixed with water and subjected to a two-step decoction process. In the first decoction, 8 times the volume of water was added, and the mixture was decocted for 2 hours. In the second decoction, 6 times the volume of water was added, and the mixture was decocted for 1.5 hours. The resulting decoction was filtered, and the filtrate was consolidated. It was then concentrated to a relative density of 1.03 ~ 1.07 (at 60°C), and 30,000 ml of water was added. The mixture was stirred, filtered, and packaged to obtain the final product.

### Sample collection and extraction

Cervical and vaginal secretions were collected from patients before and after treatment. Before sample collection, patients were provided with specific instructions, which included abstaining from smoking and alcohol for 48 hours, maintaining regular work and rest schedules, avoiding menstruation, and refraining from using suppositories, douches, or engaging in sexual intercourse. The patient was positioned in the cystotomy position, ensuring exposure of the genitals and perineum. Samples were processed as HPV DNA test sample was inserted into the cervical canal, rotated three times, left for 10 seconds, and then removed and placed into special vials for testing. Samples for TCT and HPV DNA testing were promptly sent for examination, while the cervicovaginal secretions were stored in a refrigerator at -80°C. For extraction, samples were placed in EP tubes with 400 μL of 80% aqueous methanol. After vortexing for 1 hour, the samples were centrifuged at 13,000 rpm for 10 minutes at 4°C to precipitate proteins. The supernatant was then transferred to a new tube and vacuum freeze-dried at 40°C to obtain lyophilized powder. This powder was reconstituted in 300 μL of methanol-water solution (V/V=4:1), vortexed, sonicated, and incubated at -20°C for 2 hours. After centrifugation at 4°C and 13,000 rpm for 10 minutes, 150 μL of the supernatant was transferred to an LC-MS vial for analysis.

### LC-MS analysis

Chromatographic conditions involved using a high-performance liquid chromatography-tandem high-resolution mass spectrometer Q-Exactive (Thermo) with the ACQUITY UPLC HSS T3 (2.1 × 100 mm, 1.8 μm) chromatographic column. The mobile phases consisted of phase A (0.1% formic acid in water) and phase B (0.1% formic acid in acetonitrile). The elution procedure was as follows: 2% B for 0-1.5 min, 2% B for 1.5-3 min, 2%-30% B for 3-5 min, 35%-50% B for 5-10 min, 50%-100% B for 10-15 min, and 100%-2% B for 15-16 min. The injection volume was 10 μL, and the column temperature was set at 45°C. Mass spectrometry conditions included electrospray ionization (ESI) ionization in both positive and negative ion modes, scanning range from m/z 100 to 1,500, MS1 resolution at 70,000, MS2 resolution at 17,500, capillary voltage set to 3.5 kV in positive ion mode and 3.1 kV in negative ion mode, capillary temperature maintained at 320°C, and auxiliary gas heated to 375°C with a sheath gas flow rate of 30 L/min and an auxiliary gas flow rate of 10 L/min.

### Quality control examination

To ensure data reliability and quality, 10 μL of each sample to be tested was combined to create QC samples in EP tubes. The mixture was vortexed and thoroughly mixed for 10 seconds. Subsequently, 100 μL of the QC sample was dispensed into each tube, resulting in a total of 6 tubes. The reproducibility and stability of the LC-MS system were evaluated through two consecutive injections of the QC samples initially, followed by two injections with a separation of 8 samples between them. The raw LC-MS data was converted to the mzXML file format using the Proteowizard package’s MSConvert tool. Following this, peak detection, filtering, alignment, and identification were carried out using the R xcms software package. The substances identified by XCMS were annotated with differential endogenous metabolites using the KEGG and HMDB databases. Multivariate statistical analysis techniques including Principal Component Analysis (PCA), Orthogonal Partial Least Squares Discriminant Analysis (OPLS-DA), Volcano Plot Analysis, Heatmap, and KEGG Pathway Analysis were employed to analyze and visualize the data, elucidating differential metabolites and pathways related to DaiNing Decoction intervention in HPV-infected patients.

### *Ex vivo* cellular experiments on DaiNing Decoction

The human cervical cancer cell line Hela was cultured in a complete 1640 medium supplemented with 1% penicillin and 1% streptomycin. The culture was maintained at 37°C with 5% CO2 and humidity.

#### CCK8 detection of DaiNing Decoction cytotoxicity

Hela cells were digested, counted, and seeded into a 96-well plate with 100μl of cell suspension per well, incubated overnight. Surrounding wells were filled with PBS. Cells were treated with low, medium, and high concentrations of DaiNing Decoction, each with 4 replicate wells for 24 and 48 hours. After treatment, 10μl of CCK8 working solution was added to each well and incubated for 4 hours. Absorbance at 450 nm was measured using an enzyme labelling apparatus to determine changes in cell proliferation.


Cell viability = [OD (drug) − OD (blank) / OD (undrugged) − OD (blank)] × 100%

#### Scratch experiment

Hela cells in the logarithmic growth phase were seeded into pre-labelled 6-well plates until reaching 90% confluence, then cultured with low, medium, and high concentrations of DaiNing Decoction in medium with 2% serum. Photos were taken at 0 and 24 hours, and ImageJ was used to quantify the cell migration rate and distance between the blank control and DaiNing Decoction groups.

#### Transwell assay

Hela cells in the logarithmic growth phase were seeded into the upper chamber of Trans well plates, with a medium containing 20% serum in the lower chamber. Low, medium, and high concentrations of DaiNing Decoction were added, with 3 duplicate wells for each concentration, and incubated for 24 hours. Matrigel matrix gel was prepared in the upper chamber and incubated for 48 hours. Cells were stained with crystal violet and observed under a microscope to compare cell migration or invasion between the control and DaiNing Decoction groups.

### RT-qPCR analysis

Total RNA was extracted using an RNA extraction kit, followed by determination of concentration through a series of steps including chloroform addition, column wash solution, anhydrous ethanol, and RNase-free rinse solution, along with centrifugations. Subsequently, the reverse transcription reaction system was prepared as per guidelines, and RT-qPCR was conducted using primer sequences to construct the reaction system. The resulting outcomes were documented and subjected to statistical analysis. Post-intervention with DaiNing Decoction, alterations in a-SMA, Snail, VE-Cadherin, and eNOS levels in Hela cells were observed.

### Statistical methods

Statistical analysis was performed using SPSS 21.0 statistical software. Measurement data were expressed as mean ± standard deviation (SD). One-way ANOVA was employed to compare means between groups. In instances of homogeneous variance, the SNK test and Tukey test were utilized. However, for cases of non-homogeneous variance, Tamhane’s T2 test was applied. The χ2 test was employed for analyzing categorical data. A significance level of P < 0.05 was considered statistically significant.

## Results

### Principal Component Analysis

**Figure 1:**
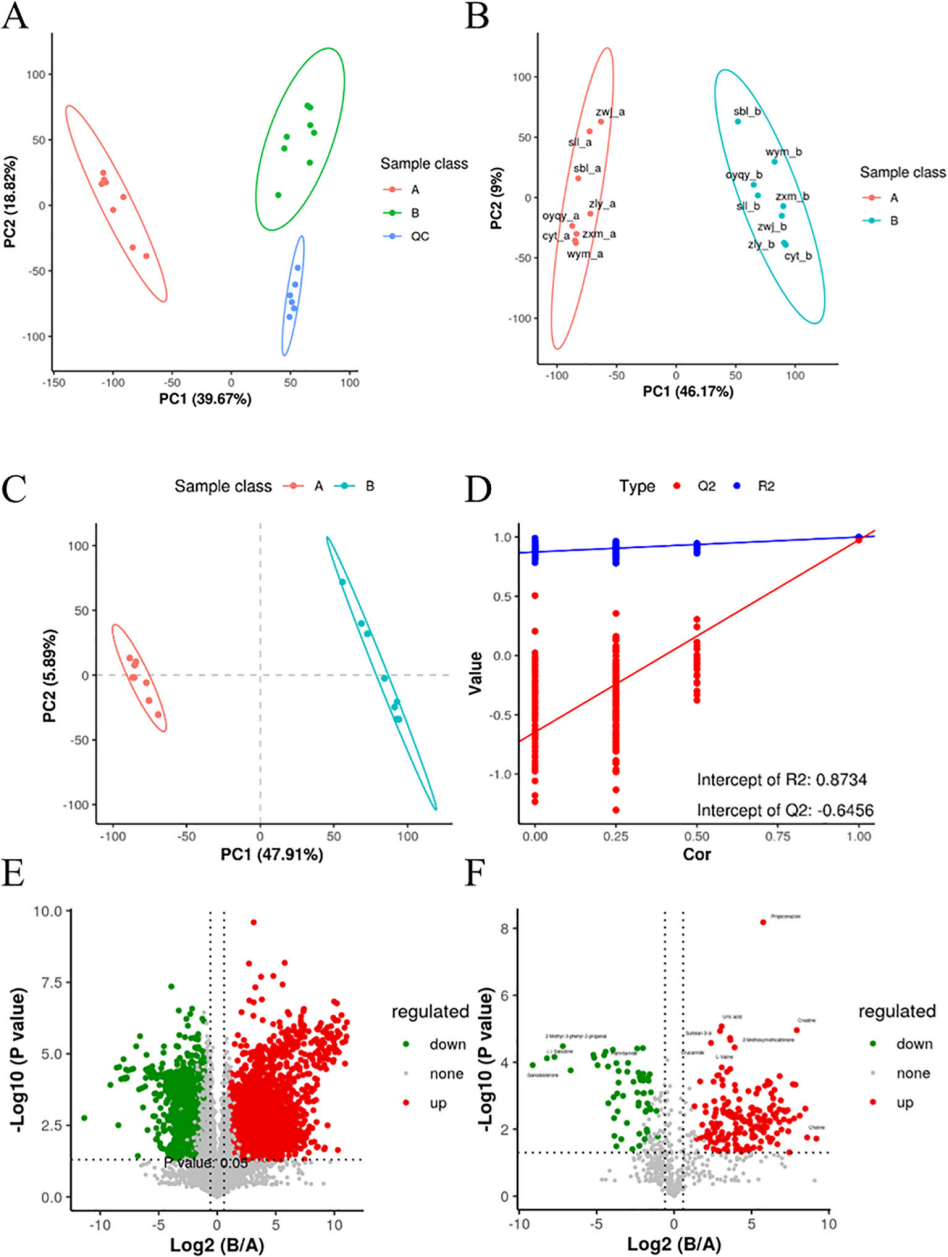
Significant differences in metabolites before and after the intervention of DaiNing Decoction. **(A, B)** Principal component analysis (PCA) **(C, D)** PLSDA model replacement test diagram. **(E, F)** The VolcanoPlot presents univariate statistical tests using volcanic plots. The ratio of the means of the intensities of the two phenotypes is shown on the abscissus, and the p value (or corrected p value) for statistical significance is shown on the ordinate and is taken as −log^10^.

Principal Component Analysis (PCA) was employed to analyze the metabolomics data obtained from both positive and negative ion patterns. This method allowed for the visualization of sample distributions, with each point on the PCA score plots representing an individual sample. The results exhibited tight clustering of quality control (QC) samples, indicating the stability and repeatability of the instrument analysis. Furthermore, a clear separation trend was observed between samples collected before and after the intervention with DaiNing Decoction (**Figure 1A**). To delve deeper into these differences, subsequent analysis using Orthogonal Partial Least Squares-Discriminant Analysis (OPLS-DA) was conducted.

#### Orthogonal Partial Least Squares-Discriminant Analysis

OPLS-DA, an unsupervised analysis method, was utilized to filter out irrelevant information and maximize group differences in metabolite profiles. The results from OPLS-DA confirmed significant differences in metabolite compositions between vaginal secretions collected before and after DaiNing Decoction intervention, corroborating the findings from PCA (**Figure 1B**).

#### Permutation test

A permutation test was conducted to assess the risk of model overfitting and the reliability of the OPLS-DA model. By subjecting the model to 200 permutation tests, it was determined that the model exhibited good adaptability and predictability, with no evidence of overfitting detected (**Figures 1C, D**).

#### Volcano plot

Volcano plots were employed to visually identify differential metabolites by representing fold changes and statistical significance. The analysis revealed significant alterations in metabolite levels before and after the DaiNing Decoction intervention, with certain metabolites exhibiting substantial changes (**Figures 1E, F**).

### Differential metabolite analysis

**Figure 2:**
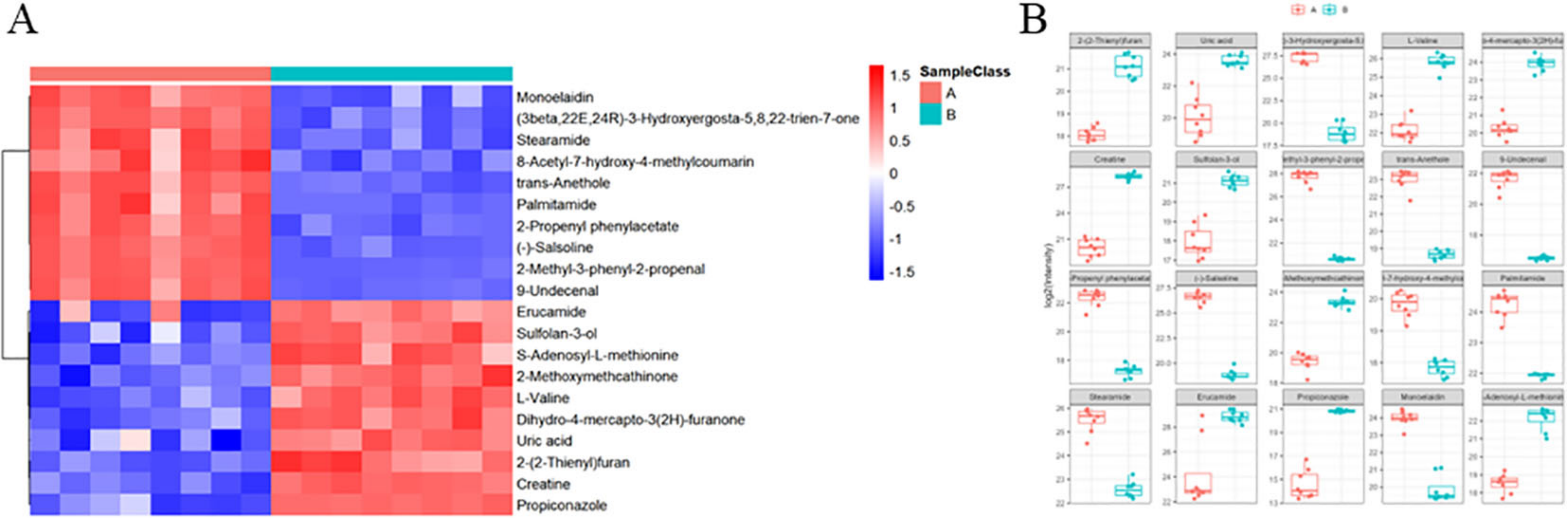
Differences metabolites heatmap. Each column has a sample, each action has a displacement peak, and the color represents the relative intensity (scale value). **(A)** Top 20 Differential Metabolites Identified Post-DaiNing Decoction Intervention. **(B)** Relative Abundance and Significance of Top 20 Differential Metabolites Post-Intervention.

Further analysis of the metabolomics data identified a set of differential metabolites that showed notable changes following the DaiNing Decoction intervention. The top twenty metabolites, ranked based on their significance, were identified and listed for detailed examination (**Figures 2A, B**). Among these, arginine and proline metabolism showed substantial alterations, with increased levels of arginine potentially supporting nitric oxide production, which is known to exhibit antiviral properties by disrupting viral replication. Additionally, glycerophospholipid metabolism was significantly affected, with metabolites such as phosphatidylcholine and lysophosphatidylcholine playing a role in membrane structure and signaling, possibly influencing viral entry and egress. Purine metabolism, particularly changes in inosine and adenosine levels, may also impact viral replication by modulating nucleotide availability. These pathways are critical for maintaining cellular homeostasis during viral infection and suggest that DaiNing Decoction may exert its therapeutic effects through modulation of key metabolic pathways involved in viral life cycles (**Figures 2A, B**).

### KEGG pathway analysis

**Figure 3:**
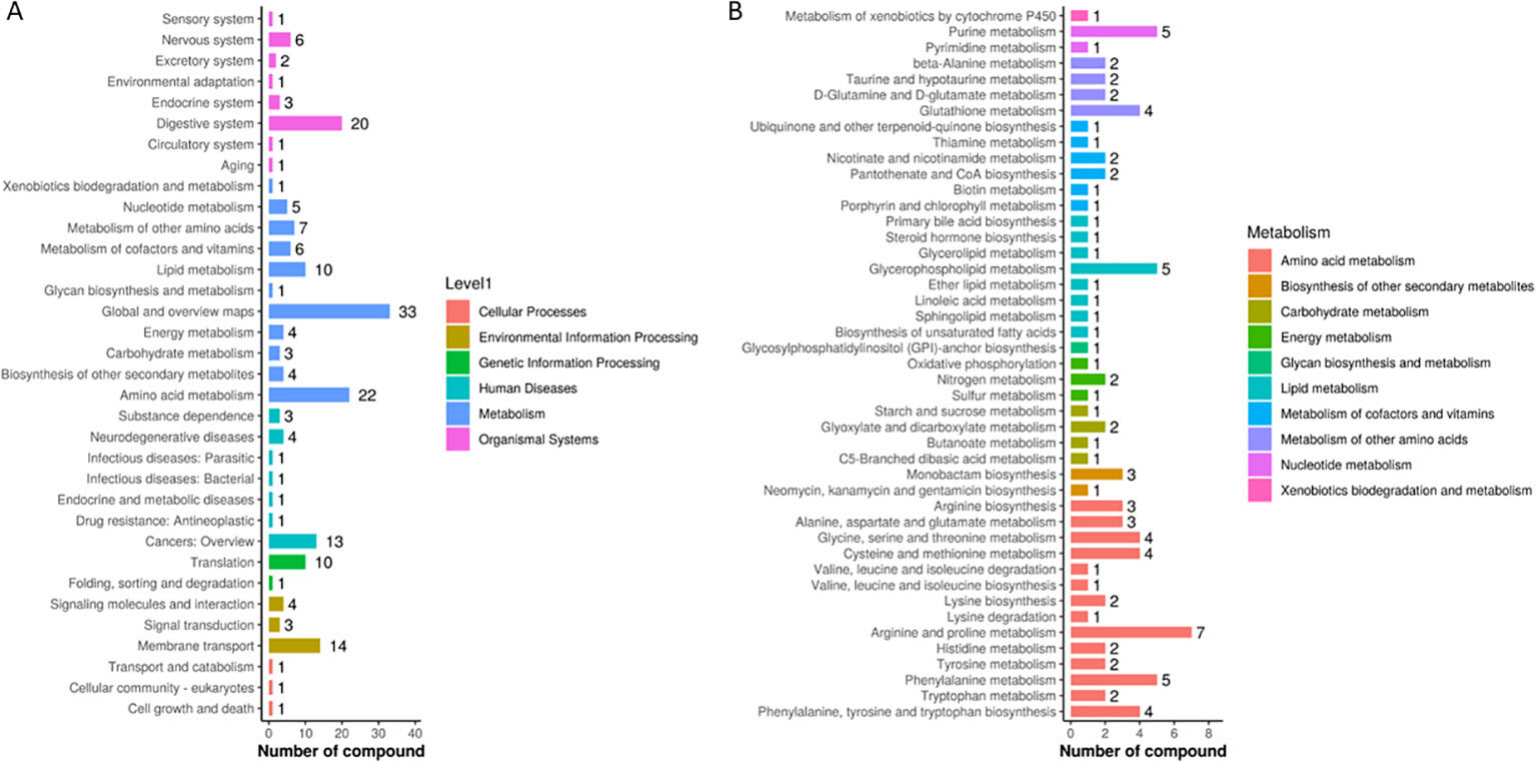
KEGG Pathway. **(A)** KEGG pathway database second level pathway statistics. **(B)** Statistics of the third level pathway under Metabolism in the second level of KEGG pathway database.

The metabolites identified through non-targeted metabolomics were analyzed using KEGG pathway analysis to elucidate the metabolic pathways influenced by the DaiNing Decoction intervention. This analysis revealed significant enrichment in pathways associated with metabolism, suggesting that the intervention induced notable metabolic alterations. Specifically, pathways involved in amino acid metabolism, lipid metabolism, and energy production were particularly affected, indicating a systemic impact of DaiNing Decoction on metabolic processes (**Figure 3A**). These findings underscore the potential of DaiNing Decoction to modulate metabolic pathways, which may contribute to its therapeutic effects in managing HPV infections.

### Metabolic pathway

The screened differential the selected differential metabolites were input into the MetaboAnalyst 5.0 online analysis website for pathway analysis, and the relevant pathways were determined with P<0.05 as the criterion. The differential metabolites between the two groups before and after the intervention of DaiNing Decoction were mainly involved in arginine and proline metabolism, purine metabolism, glycerophosphatidylcholine metabolism, and phenylalanine metabolism (**Figure 3B**).

### Cell experiment

#### CCK8 method for determining optimal drug concentration

**Figure 4:**
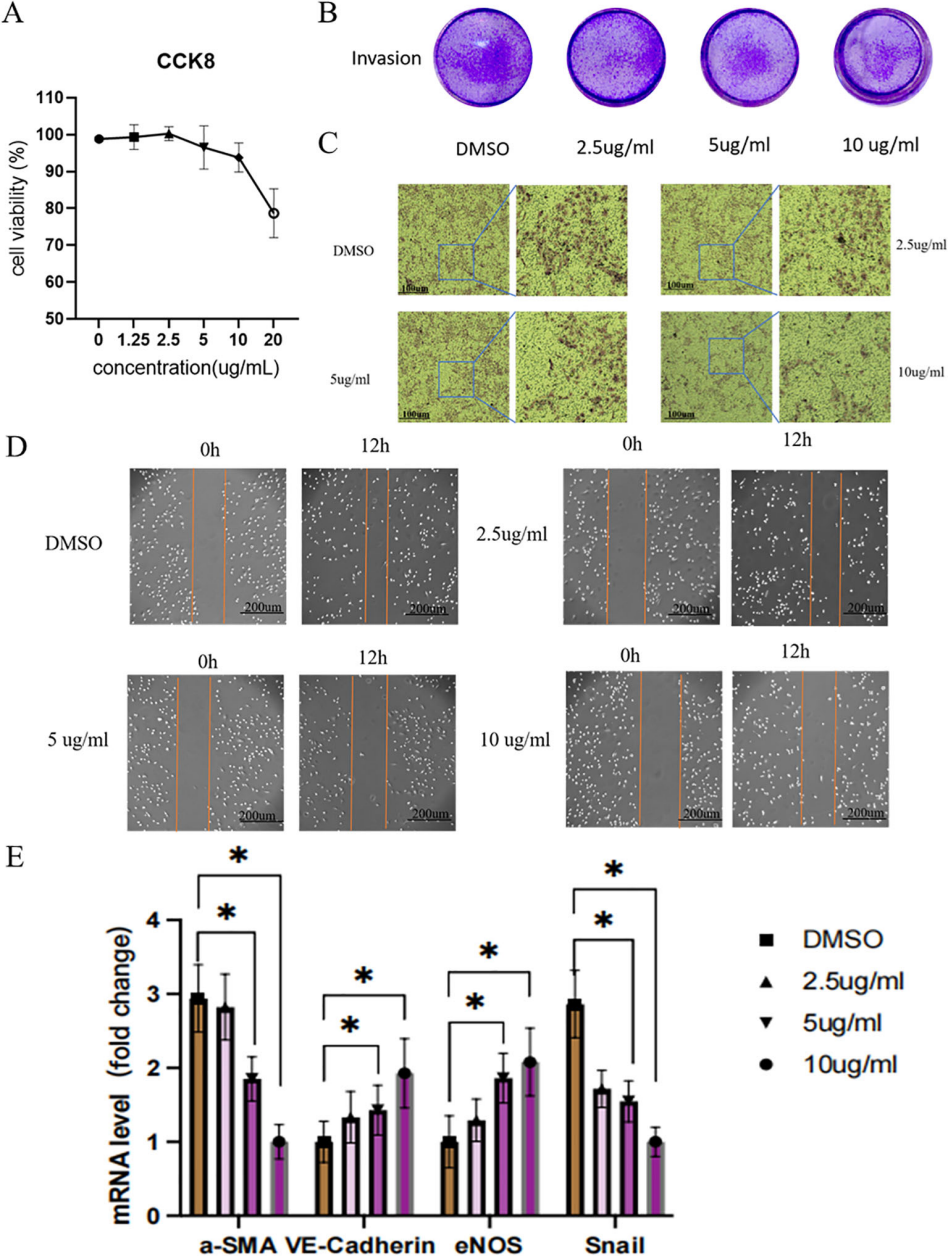
Effects of different doses of DaiNing Decoction on cell migration, invasion and EndMT.**(A)** The effect of DaiNing Decoction treatment at different concentrations on the cervical cancer cell line Hela was determined by CCK8 assay **(B, C)** Effect of DaiNing Decoction on Hela cell invasion. **(D)** The effects of different doses of DaiNing Decoction on Hela cell migration. **(E)** DaiNing Decoction regulates the mRNA level of EndMT marker (VE-Cadherin, eNOS, a-SMA and Snail) *p < 0.05.

*In vitro*, experiments were conducted to determine the optimal concentration of DaiNing Decoction for subsequent cellular assays. A range of to 0,1.25, 2.5, 5,10,20 ug/ml concentrations was tested, with careful evaluation of cell proliferation and viability. The result revealed that at 20ug/ml, the proliferation activity of the cells was significantly damaged and dead cells were observed under the microscope (**Figure 4A**). The concentrations of 2.5, 5, and 10 μg/ml were chosen for further experimentation.

#### Effect of DaiNing Decoction on Hela cell invasion

Transwell invasion assays were performed to assess the impact of DaiNing Decoction on Hela cell invasion. The results demonstrated a dose-dependent reduction in cell invasion following treatment with DaiNing Decoction, compared to the control group treated with DMSO alone (**Figures 4B, C**).

#### Effects of different doses of DaiNing Decoction on Hela cell migration

Cell scratch assays were employed to investigate the effect of DaiNing Decoction on Hela cell migration. The results indicated a dose-dependent inhibition of cell migration in response to DaiNing Decoction treatment, as evidenced by reduced migration distances compared to the control group (**Figure 4D**).

### q-PCR analysis

Quantitative Polymerase Chain Reaction (q-PCR) was performed to examine the expression levels of key genes associated with cellular processes such as cell motility and endothelial function. The results revealed significant alterations in the expression levels of a-SMA, Snail, VE-Cadherin, and eNOS in Hela cells following treatment with DaiNing Decoction (**Figure 4E**). Specifically, a-SMA and Snail expression decreased, while VE-Cadherin and eNOS expression increased, suggesting potential regulatory effects of DaiNing Decoction on these cellular processes.

## Discussion

Human Papillomavirus (HPV) encompasses a diverse group of spherical, small, non-enveloped circular double-stranded DNA viruses, primarily targeting human skin and mucosa, leading to hyperplastic lesions (Santella et al., 2022). With over 200 identified types, HPV is categorized into high-risk and low-risk types based on their carcinogenic potential (Clifford et al., 2017). Notably, HPV types 16 and 18, among the high-risk variants, are responsible for approximately 70% of cervical cancer cases (Akbari et al., 2023). Cervical cancer ranks as the second most common malignancy among women (Moore, 2006), with an alarming global annual incidence of about 600,000 cases and 340,000 deaths (Choi et al., 2022). Despite significant advances, clinical treatments, such as interferon α-2a, although effective in inhibiting viral nucleic acid replication and transcription, exhibit drawbacks including a high recurrence rate post-drug withdrawal and adverse reactions (Dusheiko, 1997; Hietanen et al., 1998). DaiNing Decoction, a traditional Chinese herbal remedy, comprises Radix Millettiae Speciosae, Cyrtomium Fortunei, Smilacis Chinae Rhizoma, ginkgo seeds, and Cockscomb. Radix Millettiae Speciosae and *Cyrtomium Fortunei* possess anti-tumour, anti-malaria, and anti-viral properties. Meanwhile, Smilacis Chinae Rhizoma combined with ginkgo seeds is known for its dampness and phlegm-removing effects, commonly employed in tumour disease treatment. Epithelial-mesenchymal transition (EMT) represents a pivotal developmental process facilitating the migration of adherent epithelial cells (Manfioletti and Fedele, 2023). During EMT, epithelial cells lose their polarity, undergo morphological and structural changes, and relinquish typical epithelial characteristics (Mittal, 2018). Subsequently, cells transition into mesenchymal cells, acquiring migratory and invasive properties, facilitating the progression of early tumors into invasive malignancies (Zhang and Weinberg, 2018). Given its role in enhancing cell mobility, EMT emerges as a significant player in tumour progression (Taki et al., 2021). Quantitative Polymerase Chain Reaction (qPCR) analysis revealed that DaiNing Decoction treatment significantly reduced the expression of α-SMA and Snail while upregulating the expression of VE-Cadherin and eNOS, particularly at higher doses (P<0.05). α-SMA, an actin variant, participates in cytoskeletal remodeling, promoting EMT in Hela cells, thereby influencing tumour cell invasion and metastasis (Hu et al., 2020). Unlike treatments like interferon α-2a, which target viral replication but have limited impact on tumour progression, DaiNing Decoction appears to directly influence the molecular mechanisms involved in EMT, a critical factor in cancer metastasis. Specifically, it upregulates VE-Cadherin and eNOS while downregulating α-SMA and Snail, markers associated with epithelial cell adhesion and migration. This dual modulation contrasts with conventional therapies, which primarily focus on inhibiting viral replication rather than addressing tumour cell invasion. Additionally, DaiNing Decoction’s unique combination of herbal compounds may provide a broader, multi-targeted inhibition of EMT, potentially offering a complementary strategy alongside current treatments (Farzanehpour et al., 2020; Bereczki-Temistocle et al., 2023). These findings suggest that DaiNing Decoction inhibits Hela cell migration and invasion by modulating the expression of EMT-related molecular markers. Proline metabolism plays a dual role in cancer, linking apoptosis to cancer cell survival and metastasis. Metabolomics analysis revealed that the metabolism of arginine and proline represents a crucial pathway in the differential metabolite profiles before and after the DaiNing Decoction intervention. Serine-arginine protein kinase-1 (SRPK1), a precursor mRNA splicing factor, plays a role in promoting EMT progression (Duggan et al., 2022). Proline, a significant secondary proteinogenic amino acid, exhibits dual roles in cancer, linking apoptosis to cancer cell survival and metastasis (Phang et al., 2010). Furthermore, the DaiNing Decoction intervention showed associations with purine metabolism, glycerophosphatidylcholine metabolism, and phenylalanine metabolism. HPV-positive cells display elevated levels of purine derivatives to support nucleic acid production and growth (Pappa et al., 2021). Moreover, decreased phenylalanine levels in cervical tissues of patients with cervical squamous cell carcinoma suggest its potential as a screening marker (Chakravarthy et al., 2022). Despite the insightful findings, our study is limited by the absence of *in vivo* animal experiments and the lack of investigations involving mass spectrometry analysis of DaiNing Decoction constituents and quantitative analysis of its effective components. However, our study implies that DaiNing Decoction may inhibit Hela cell invasion and migration by modulating EMT via upregulating VE-Cadherin and eNOS expressions and downregulating α-SMA and Snail expressions. Since herbal extracts can vary based on factors like geographic source, cultivation conditions, and extraction methods, the consistency of active components may fluctuate. This poses challenges in standardizing the decoction for clinical application and replicating results. Furthermore, the absence of quantitative mass spectrometry analysis of the active compounds in DaiNing Decoction limits the ability to pinpoint the exact molecular constituents responsible for its anti-tumour and anti-viral effects. Addressing these factors in future research is critical to ensuring reliable therapeutic outcomes.

## Conclusion

Our study highlights the significant therapeutic potential of DaiNing Decoction in inhibiting HeLa cell migration and invasion through the modulation of epithelial-mesenchymal transition (EMT). By upregulating the expressions of VE-Cadherin and eNOS while downregulating α-SMA and Snail, DaiNing Decoction demonstrates promising anti-cancer properties, particularly in the context of cervical cancer. While the absence of *in vivo* animal studies limits our understanding of its full therapeutic profile, our findings lay the groundwork for future clinical applications of this herbal formula in HPV treatment. Further research, including mass spectrometry analysis of its constituents and quantitative assessment of its active components, will be crucial to ensuring consistency and understanding its mechanisms of action. Despite these limitations, our results suggest that DaiNing Decoction could emerge as a viable complementary therapy in cancer management, opening new avenues for integrating traditional Chinese medicine into modern oncology practices.

## Data Availability

The original contributions presented in the study are included in the article/supplementary material. Further inquiries can be directed to the corresponding authors.
